# A machine learning pipeline to classify foetal heart rate deceleration with optimal feature set

**DOI:** 10.1038/s41598-023-27707-z

**Published:** 2023-02-13

**Authors:** Sahana Das, Sk Md Obaidullah, Mufti Mahmud, M. Shamim Kaiser, Kaushik Roy, Chanchal Kumar Saha, Kaushik Goswami

**Affiliations:** 1 West Bengal State University, Kolkata, 700126 India; 2 Aliah University, Kolkata, 700156 India; 3 Department of Computer Science, Nottingham Trent University, Nottingham, NG11 8NS UK; 4 Jahangirnagar University, Savar, Dhaka 1342 Bangladesh; 5 Biraj Mohini Matri-Sadan & Hospital, Kolkata, 700126 India; 6 Tata Consultancy Services, Kolkata, 700156 India

**Keywords:** Computational science, Information technology

## Abstract

Deceleration is considered a commonly practised means to assess Foetal Heart Rate (FHR) through visual inspection and interpretation of patterns in Cardiotocography (CTG). The precision of deceleration classification relies on the accurate estimation of corresponding event points (EP) from the FHR and the Uterine Contraction Pressure (UCP). This work proposes a deceleration classification pipeline by comparing four machine learning (ML) models, namely, Multilayer Perceptron (MLP), Random Forest (RF), Naïve Bayes (NB), and Simple Logistics Regression. Towards an automated classification of deceleration from EP using the pipeline, it systematically compares three approaches to create feature sets from the detected EP: (1) a novel fuzzy logic (FL)-based approach, (2) expert annotation by clinicians, and (3) calculated using National Institute of Child Health and Human Development guidelines. The classification results were validated using different popular statistical metrics, including receiver operating characteristic curve, intra-class correlation coefficient, Deming regression, and Bland-Altman Plot. The highest classification accuracy (97.94%) was obtained with MLP when the EP was annotated with the proposed FL approach compared to RF, which obtained 63.92% with the clinician-annotated EP. The results indicate that the FL annotated feature set is the optimal one for classifying deceleration from FHR.

## Introduction

Monitoring of labour is essential as there is a chance that the fetus might suffer from oxygen deficiency which ultimately may lead to lifelong debility or even death. A major source of information about foetal health is Cardiotocography (CTG), which concurrently records Foetal Heart Rate (FHR) and the mother’s uterine Contraction Pressure (UCP). Physicians visually evaluate the patterns of these two signals and apply the knowledge of their prior experience to evaluate the status of foetal health and to take appropriate actions. Since there is a great disparity in how physicians interpret the signals, there are, at times, false alarms that lead to unnecessary C-sections. On the other hand, sometimes significant, ominous patterns are overlooked, resulting in foetal compromise. 50% of birth-related brain damages are avoidable with accurate interpretation of CTG^1^. A huge legal cost is involved due to the malpractice claims that are filed every year^2^. This is also evident from the statistics reported between 2005 and 2014 that in the US, Obstetrics and Gynaecology claims had the second-highest average indemnity payment and the fifth-highest paid-to-closed ratio of all medical specialities^2^. Out of the four parameters of FHR, deceleration is the most complex to interpret. It is also central to the correct interpretation of CTG, and hence the foetal status^3^. Emphasis is placed on the association between the correct physiology of deceleration and the patterns of FHR and UCP changes in order to identify the foetal status. Decelerations are generally not visible in antenatal CTG. However, if present, then foetal health should be further investigated. Mild deceleration usually requires no intervention, but during labour, abrupt and frequent dips of FHR from the baseline with varying depth and duration may be ominous. Standard guidelines for CTG interpretation put forward by the National Institute of Child Health and Human Development (NICHD), the International Federation of Gynaecology and Obstetrics (FIGO), the Royal College of Obstetricians and Gynaecologists (RCOG) etc., classify deceleration based on the shape or time descent of the FHR^4–6^. Decelerations are categorised as ‘early’, ‘late‘ and ‘variable’. These categorisations are mainly based on the temporal relationship between the deceleration, its duration and the corresponding uterine contraction and the duration of contraction. ‘Early’ decelerations are considered benign, while ‘late’ and ‘variable’ decelerations are considered ‘pathological’ and ‘suspicious’ respectively; hence these two decelerations require careful attention to ensure foetal good health.

Despite the existence of several guidelines, disagreement arises in the classification of deceleration. A survey revealed that British practitioners considered ‘early’ deceleration as the most common, while NICE guidelines 2007 reported that ‘early’ decelerations are the rarest and the ‘variable’ decelerations are most common^7^. When it comes to the classification of deceleration, it is important to relate the deceleration nadir (i.e., the lowest point in the declaration) with the peak of the contraction. According to the literature, the ‘early’ deceleration occurs when the two points match. This is not a very common phenomenon. Deceleration is classified as ‘late’ if it starts after the peak of the uterine contraction. Nadir is thus reached almost at the end of the contraction.

True ‘early’ decelerations whose nadir coincides exactly with the peak of the contraction is rare. It would be wrong to classify decelerations as ‘late’ that start recovering immediately after the peak of the contraction. In such cases, hard classification boundaries are not appropriate. Fuzzy classification is thus more appropriate for such borderline cases.

### Physiology of FHR deceleration

FHR deceleration is the transient drop in the heart rate below the baseline value by 15 bpm or more and lasting for 15 s or longer. There exists a temporal relationship between decelerations and uterine contraction, which in turn is linked with rising in the internal pressure of the uterus and a decrease in maternal uterine artery blood flow. Even in normal labour, placental gas exchange is reduced. This leads to a fall in pH and oxygen tension and elevation of CO2, and base deficit in normal labour.

For most fetuses, the placental oxygen capacity is enough to overcome the repeated reduction in oxygen supply during labour. However, for fetuses that are already vulnerable, this repeated hypoxia may become life-threatening. It was also found that there are times when even a normal fetus is not able to withstand uterine hyperstimulation^8^.

Asphyxia is the deficiency of oxygen which, if prolonged, leads to hypoxemia and subsequent metabolic acidosis or accumulation of the waste product in the blood. Most hypoxic episodes during labour are brief and benign, lasting less than 1 min. These are reflected by brief deceleration. However, if hypoxia is severe and lasts more than three minutes, the initial vagal bradycardia is sustained by myocardial hypoxia. Thus the depth of deceleration is associated with a reduction in uteroplacental blood flow^9^. Studies have shown that deep deceleration is associated with an intense lack of oxygen to the brain with a chance of neuronal injury if the hypoxemia lasts more than ten minutes. Whether decelerations of shorter duration are benign or not depends upon three factors:

- Criticality of foetal health before labour.
- Pre-labour placental reserve of oxygen
- Duration and frequency of deceleration

**Figure 1:**
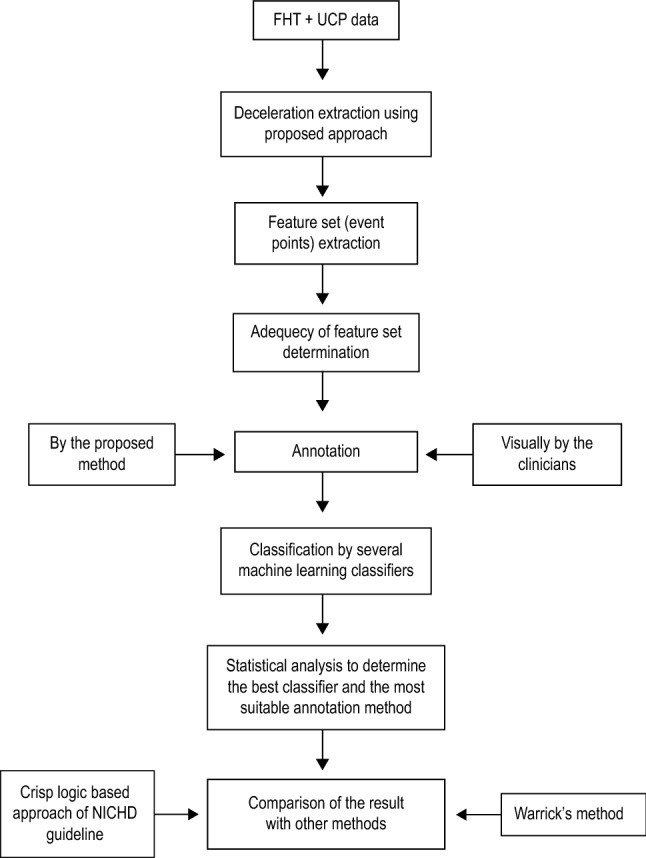
Flow diagram depicting the overview of the proposed model.

Different obstetric bodies, such as NICHD, FIGO etc., provided standard guidelines for the classification of deceleration based on its shape, time and duration with respect to the uterine contraction. The overall process overview of the proposed work is shown in Fig. 1. The details of this categorisation are shown in Table 1.

**Table 1:** Categorisation of the deceleration of FHR.

Type of declaration	Stage of labour	Nadir of declaration	Physiology	Clinical opinion
Early	1st or 2nd	Peak of uterine contraction	Head compression	Benign
Late	Any	$> 30$ s after the peak of the contraction	Foetal hypoxia	Pathological
Variable	Any	Variable	Cord compression	Suspicious/Pathological

**Figure 2:**
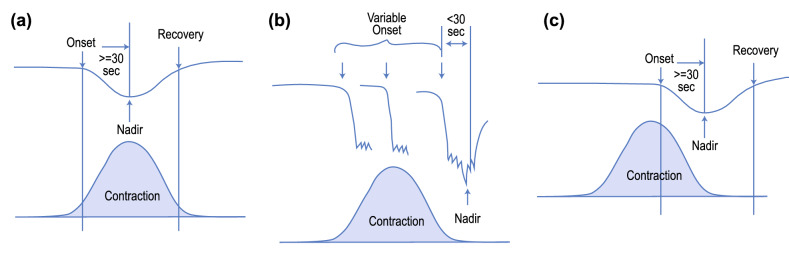
(**a**) Early Deceleration—the peak of the contraction coincides with the nadir of the deceleration, (**b**) Variable Deceleration—the nadir of the deceleration can occur anywhere during the contraction, and (**c**) Late Deceleration—the nadir of the deceleration coincides with the end of the contraction.

The three types of decelerations and their temporal relationships with uterine contractions are shown in Fig. 2.

#### Physiology of deceleration types

**Figure 3:**
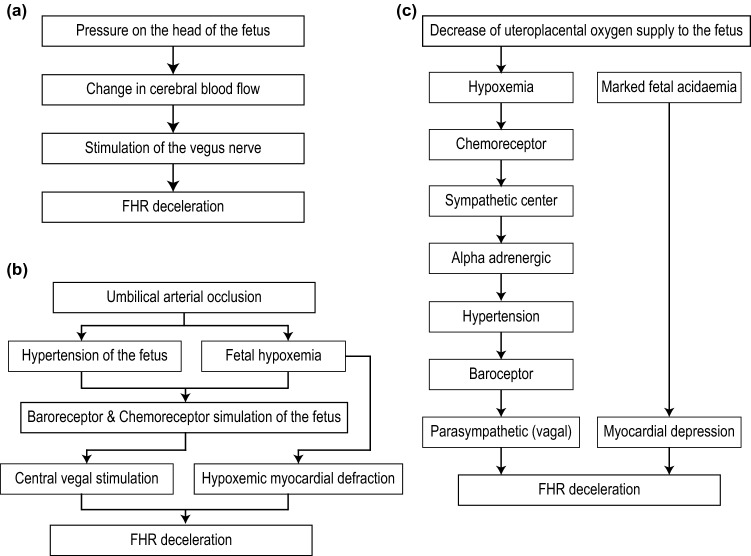
Physiology of (**a**) Early deceleration, (**b**) Variable deceleration, and (**c**) Late deceleration.

In Early deceleration, all the event points of the deceleration and the corresponding uterine contraction coincide. In Late deceleration, the peak of the uterine contraction is reached before the start of the deceleration, and the uterine contraction ends before the deceleration reaches its nadir. Variable deceleration does not have any particular temporal and spatial relationship with uterine contraction. The physiology of early deceleration is shown in Fig. 3a–c.

### Problems with the identification and classification of deceleration

In any developed country, the most commonly used method of foetal monitoring is by CTG. But there are many flaws in the interpretation. This is evident from the lawsuits faced by obstetricians in the UK. NHS had to pay GBP3.1 billion related to maternity care in the last decade. Most of these cases were due to cerebral palsy and errors in interpreting CTG^7^. The interpretation of foetal CTG is considered to be one of the most controversial and problematic issues in Obstetrics due to human error, incorrect usage of certain medications and frequent contamination of foetal CTG with maternal CTG^10^. Therefore, the classification of deceleration plays a major role in the classification of FHR patterns into the three-tier system, i.e., ‘normal’, ‘suspicious’ and ‘pathological’. Intrapartum foetal surveillance and the interpretation of CTG not only require a thorough understanding of foetal physiological response to hypoxia but also the skill to recognise numerous patterns and the ability to incorporate the knowledge with each clinical case^10^.

Although there are three different classifications of deceleration—early, late, and variable, the exact definition of each type and their medical implication vary from time to time and from country to country. For decades, most clinicians in the UK classified deceleration as ‘early’ if it started with the Uterine Contraction (UC) and ended before the end of the contraction, irrespective of the descent. As per NICHD guideline the minimum duration of an deceleration from start to nadir is 30 s but for the sake of clarity and easy screening we have considered 15 s as minimum duration of possible deceleration in the first phase of screening.

In 2007 NICE modified its guideline based on the categorisation of deceleration from the work of Hon^11^. According to Hon, the main criteria for categorising deceleration is the ‘time of descent’, irrespective of the relationship to contraction. For ‘early’ and ‘late’ decelerations, the ‘time of descent’ is gradual, while for ‘variable’ deceleration, it is rapid. It also specifies that the ‘early’ and ‘late’ decelerations are uniform in shape. As a result, in recent times, decelerations are mainly categorised as ‘variable’ in both UK and USA. This definition was included not only in the guidelines but also in online CTG training modules like EFM.

All rapid decelerations, as a result, were categorised as ‘variable’ even though most of them started during the start of the contraction and the nadir corresponded to the peak of the contraction. Sholapurkar argued that this is not a robust method of categorisation of deceleration. ‘Truly uniform’ and ‘gradual’ shape of early and late decelerations are practically non-existent^12^.

Also, the term ‘repetitive’ is misinterpreted as decelerations occurring with all contractions. This is another reason for failing to identify ‘early’ and ‘late’ decelerations. Since all head compressions do not cause decelerations, ‘early’ deceleration, if present, can be linked with maximum but not all contractions. Sholapurkar thus argues that the term ‘repetitive’ should be replaced by the term ‘recurrent’ as is done by NICHD. Recurrent means associated with more than 50% of contractions in any 20 min segment. The clue to a benign reflex (early deceleration) against the pathological nature of deceleration (late/variable) lies in timing with respect to the uterine contractions rather than on the slope of the descent. Due to such varied opinions about the classification of deceleration, it becomes difficult to classify the CTG in one of the three categories accurately. Ultimately it leads to a high false positive rate of diagnosis.

Visual analysis is the common method of diagnosis; however, the estimation made in such scenarios is based on the clinician’s intuition. According to Ham^13^, intuition is determined by the clinician’s experience, domain knowledge, and logical thinking. They subconsciously employ pattern recognition skills to analyse the visual information to make an estimation. They match the situation with some previous experience to reach a conclusion, which might lead to an inaccurate estimation. When a second clinician is presented with the same clinical evidence, an altogether different diagnosis may be obtained due to the difference in their skill and knowledge, thus, giving rise to intra-observer variation^14^.

### Related work

Several researchers over the years have proposed soft-computing-based decision-making models to address these issues. The terms periodic and episodic decelerations to identify the patterns that coincide with the uterine contractions and those that occur irrespective of uterine contractions, respectively, coined by Jezeweski et al.^15^. They used MLP to distinguish between the two with an accuracy of 93%. The peak and nadir of the uterine contraction and the deceleration, respectively, were detected by Warrick et al.^16^ and used ANN to classify the deceleration with an accuracy of around 79%. An 8-layer deep Convolution Neural Network (deep-CNN) was used to detect foetal acidemia by Zhao et al.^17^ with an accuracy of 98.3%. An ANN-based model to classify the CTG with 92.4% accuracy was proposed by Comert et al.^18^. Foetal acidemia was predicted by the expert system designed by Czabanski et al.^19^ using weighted fuzzy scoring and least square SVM. The performance accuracy of the system was 92%. Deep-adaptive neuro-fuzzy inference system (deep-ANFIS) was used for the overall classification of CTG with an accuracy of 96.8%^20^. Hidden Markov Model (HMM) exhibited an accuracy of 84.7% in identifying potentially compromised fetuses during the antepartum period^21^. CNN was used on the CTU-UHB dataset to classify the FHR pattern with an accuracy of 98.34%^17^. Generative Model-based evaluation to categorise the FHR signal yielded a weighted relative accuracy (WRA) of just 0.425^22^. Some of the currently available commercial systems are SonicAid FetalCare^23^, NST-EXPERT^24^, OmniView SisPorto 3.5^25^, PeriCALM^26^ etc. Though these systems extract the features of FHR automatically, the clinicians do the final analysis visually.

Accurate identification and classification of deceleration is an important indicator of foetal health and for the overall classification of CTG. A robust algorithm for the classification of deceleration was not found in any of the published literature. The novelty of the proposed model is:

- Use of fuzzy logic-based method to estimate the length and width of the negative deviations from the baseline to identify the true deceleration.
- Computes the event points of both FHR and the corresponding uterine contraction using the fuzzy logic-based approach.
- Classification of the deceleration as Early, Late, and Variable using various machine learning algorithms. The results were compared with the classification done with the crisp-logic-based method provided in the NICHD guideline and the NN-based model proposed by Warrick.

## Methods

We have used the CTU-UHB (Czech Technical University - University Hospital in Brno) dataset for this work^27^. which is downloadable from this link: https://physionet.org/content/ctu-uhb-ctgdb/1.0.0/. This dataset comprises 552 intrapartum CTG records collected between 2010 and 2012 at UHB. The CTG records were carefully selected from 9164 recordings and were sampled at 4 Hz. We considered 125 traces with over 37 weeks of gestation.

Identification of deceleration is dependent on the estimation of baseline of FHR (BL), which is calculated as beats-per-minute (bpm) using a previously proposed algorithm^28^. However, to calculate the baseline, first the accelerations and decelerations are to be removed from the signal. But the correct identification of these events is dependent on the baseline. To overcome this deadlock situation, we have used an recursive algorithm from a previous work^29^ . After the estimation of BL, the fuzzy membership values are used to the compute decelerations.

This algorithm estimated the deceleration, assessed the width and amplitude of any negative deviation from the baseline, and identified it as deceleration if both the amplitude and the width conform to the definition provided by the different international obstetric bodies. Every deceleration, *De*, was identified using three points—(1) the beginning (where the foetal heart rate crosses the baseline), the nadir of *De*, and the end (where FHR again crosses the baseline). The duration of each *De* was noted.

Each FHR data is represented using *m* data points of $F= {f_1, f_2, \ldots , f_m}$. As data is traversed from left to right, considering there are *r* deceleration segments, each segment $P_i= {p_1, p_2, \ldots , p_n}$ with $i=1, 2,\ldots , r$, is encountered within the baseline limits. That is, for deceleration *De*, $p_1 \le BL, p_2,\ldots .,p_{n-1} < BL,$ and $p_n \ge BL$. Here the time periods are measured in seconds with $p_1$ occurring at time $t_1$ and $p_n$ occurring at time $t_n$. The nadir of the deceleration, $p_{min}$ and its corresponding time $t_{min}$ are identified as the $min({p_1, \ldots , p_n})$ when the segments lie with the bound such that $15 \le (t_n-t_1)<600$. A segment $P_r$,$r\in i$ of FHR is considered a deceleration if $(BL-p_{min}) \ge 15$ bpm and $t_n-t_1 \ge 15$ s.

### Algorithm for determining deceleration

There are twelve event points—the beginning point of deceleration ($D_{st\_point}$), the nadir of the deceleration ($D_{n\_point}$), the endpoint of deceleration ($D_{e\_point}$), the time at which the deceleration starts ($D_{st\_time}$), time the deceleration reaches the nadir ($D_{n\_time}$), end time of the deceleration ($D_{e\_time}$), the start point of UCP ($U_{start}$), the peak point of UCP ($U_{peak}$), the endpoint of UCP ($U_{end}$), the start time of UCP ($U_{st\_time}$), the peak time of the UCP ($U_{p\_time}$), end time of UCP ($U_{e\_time}$). These can be written as six tuples as follows:

1. ($D_{st\_time}$, $D_{st\_point}$), ($D_{n\_time}$, $D_{n\_point}$), ($D_{e\_time}$, $D_{e\_point}$) are associated with deceleration.
2. ($U_{st\_time}$, $U_{start}$), ($U_{p\_time}$, $U_{peak}$), ($U_{e\_time}$, $U_{end}$) are associated with UCP corresponding to the deceleration.

#### Algorithm 1: estimation of deceleration event points

The definition of deceleration provided by different international bodies provides strict measurement criteria without providing a means of identifying the signal segments that lie at the boundary in terms of the width and the depth. We thus propose a fuzzy-logic-based method to identify a signal segment as a deceleration of FHR and thus estimate the event points.
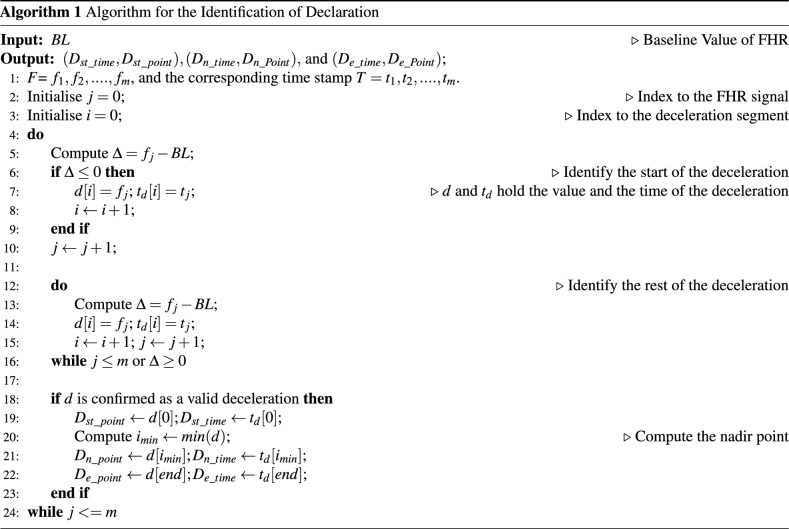


#### Fuzzification and detection of identifiable deceleration

Due to the recent increase of fuzzy logic-based methods in diverse applications^30–35^, we used a fuzzy logic-based approach to identify the length and width of the detected events to define a period with negative deviation from baseline as deceleration. Features (e.g., Duration, **T** and Depth, **N**) and their corresponding membership functions are listed in Table 2. A 2-input fuzzy model has been designed with 16 rules (see Table 3) which were obtained after consultation with the clinicians. According to the NICHD guidelines, the deceleration length should be at least 15 s but not more than 10 min, which is considered a baseline change. The difference between the nadir and the baseline should be at least 15 bpm for it to be considered to be a deceleration. However, clinical scenarios may not conform to such strict definitions.

**Table 2:** Membership functions of the features of deceleration^14^.

Feature	Description	Membership function (mf)
Duration (T)	Duration of time FHR is below the baseline	Trapezoidal
Depth (N)	Distance of the nadir from the baseline	Trapezoidal

Defuzzification is done with the help of a neural network (NN)^30,36^. To avoid possible bias, we used 5-fold cross-validation. The training process was applied to all the folds except one used for testing. The obtained output is the binary classification with 0 and 1 indicating the absence or presence of identifiable deceleration.

**Table 3:** Fuzzy-Rulebase for the identification of deceleration

ANT									CON
T<13.5	13.5 $\le$ T<15	15$\le$ T $\le$ 120	120 < T $\le$ 360	360 < T < 600	T $\ge$ 600	N < 12	12 $\le$ N < 15	N $\ge$ 15	
$\checkmark$	$\times$	$\times$	$\times$	$\times$	$\times$	$\times$	$\times$	$\times$	**ND**
$\times$	$\times$	$\times$	$\times$	$\times$	$\times$	$\checkmark$	$\times$	$\times$	**ND**
$\times$	$\checkmark$	$\times$	$\times$	$\times$	$\times$	$\checkmark$	$\times$	$\times$	**ND**
$\times$	$\checkmark$	$\times$	$\times$	$\times$	$\times$	$\times$	$\checkmark$	$\times$	**ND**
$\times$	$\checkmark$	$\times$	$\times$	$\times$	$\times$	$\times$	$\times$	$\checkmark$	**ND**
$\times$	$\times$	$\checkmark$	$\times$	$\times$	$\times$	$\checkmark$	$\times$	$\times$	**ND**
$\times$	$\times$	$\checkmark$	$\times$	$\times$	$\times$	$\times$	$\checkmark$	$\times$	**ND**
$\times$	$\times$	$\checkmark$	$\times$	$\times$	$\times$	$\times$	$\times$	$\checkmark$	**D**
$\times$	$\times$	$\times$	$\checkmark$	$\times$.	$\times$	$\checkmark$	$\times$	$\times$	**ND**
$\times$	$\times$	$\times$	$\checkmark$	$\times$	$\times$	$\times$	$\checkmark$	$\times$	**ND**
$\times$	$\times$	$\times$	$\times$	$\checkmark$	$\times$	$\checkmark$	$\times$	$\times$	**ND**
$\times$	$\times$	$\times$	$\times$	$\checkmark$	$\times$	$\times$	$\checkmark$	$\times$	**PD**
$\times$	$\times$	$\times$	$\times$	$\checkmark$	$\times$	$\times$	$\times$	$\checkmark$	**PD**
$\times$	$\times$	$\times$	$\times$	$\times$	$\checkmark$	$\checkmark$	$\times$	$\times$	**ND**
$\times$	$\times$	$\times$	$\times$	$\times$	$\checkmark$	$\times$	$\checkmark$	$\times$	**BC**
$\times$	$\times$	$\times$	$\times$	$\times$	$\checkmark$	$\times$	$\times$	$\checkmark$	**BC**

**ANT**: Antecedent; **CON**: Consequent; **ND**: Not Deceleration; **D**: Deceleration; **PD**: Prolonged Deceleration; **BC**: Baseline Change.

### Classification of deceleration

#### Feature sets

Three obstetricians of various levels of experience were involved in the study. They studied the CTG traces independently and marked the beginning, end, and nadir. Depending upon the temporal and spatial location of the corresponding uterine contractions and decelerations, they labelled the deceleration as early, late, or variable. The expert consensus did the final annotation to avoid any bias. We have created the following feature sets:

1. First feature set S1 consists of the event points estimated using our proposed method, the value of the baseline estimated using an existing algorithm^37,38^, and the classification label provided by the clinicians.
2. The second feature set, S2, consists of the event points, the baseline, and the label marked by the clinicians.

Both data sets were used as input to several classifiers such as Random Forest, Multilayer Perceptron (MLP), FURIA, and Simple Logistics. Their performances were compared using several statistical estimation techniques. We have also estimated the crisp logic-based classification given in the NICHD guideline and compared the result with the label provided by the clinicians using statistical methods. The tuples associated with UCP are computed likewise, taking the basal value of UCP as zero.

#### Adequacy of the feature set

**Figure 4:**
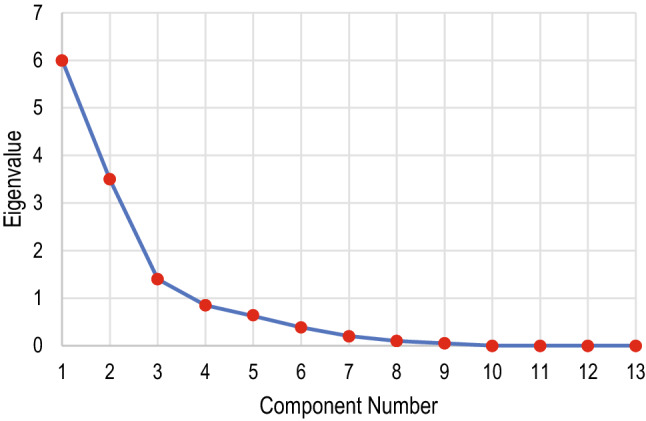
Screen plot showing the number of relevant components and their eigenvalues in decreasing order. Since the eigenvalue dropped stiffly, any additional feature would add little to the existing information.

**Figure 5:**
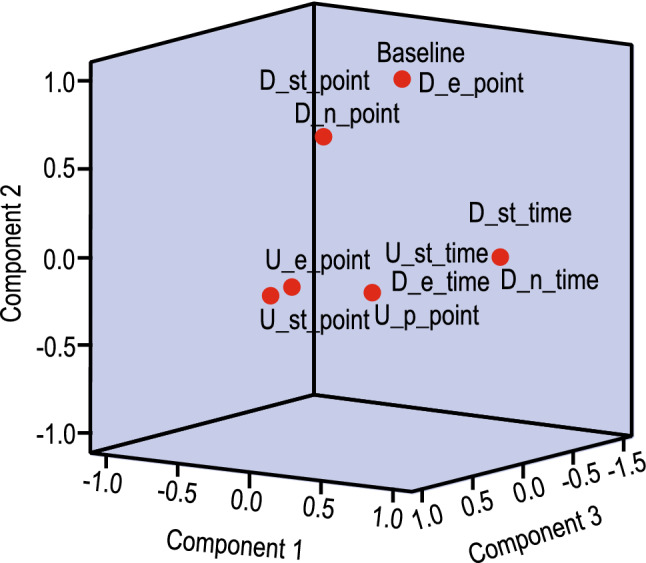
Component plot in rotated space showing the amount of correlation among the features. D_e_time is highly correlated with U_p_point, but no correlation exists between Baseline and D_n_point.

We have taken into consideration a total of 13 features. The optimality of the feature set was confirmed using Kaiser-Meyer-Olkin (KMO)^14^ and Bartlett’s test, as shown in Table 4. The component matrix is given in Table 5 extracted using Principal Component Analysis (PCA). The Scree plot and the Component plot are given in Figs. 4 and 5, respectively.

**Table 4:** Kaiser-Meyer-Olkin (KMO) test to measure the adequacy of the feature set. Bartlett’s test of sphericity tests that the correlation matrix is the identity matrix.

KMO measure of sampling adequacy		0.815
Bartlett’s test of sphericity	Approx. $\chi ^2$	3234.897
	df	78
	Sig.	0.000

**Table 5:** Component matrix to show the correlation between the features and the class.

	Component		
	1	2	3
U_st_time	0.983		
D_n_time	0.983	$-0.102$	
D_st_time	0.982	$-0.102$	
D_e_time	0.982	$-0.104$	
U_e_time	0.954		
U_p_time	0.923		
U_p_point	0.416	$-0.391$	0.362
D_st_point	0.198	0.945	0.104
Baseline	0.241	0.935	0.163
D_e_point	0.254	0.932	0.170
U_e_point		$-0.436$	0.694
U_st_point	$-0.140$	$-0.449$	0.587
D_n_point		0.485	0.566

### Comparison with other methods

#### Algorithm 2: NICHD guideline based estimation and classification of deceleration

The third feature set, S3, consists of event points, baseline and the label computed using the crisp-logic-based method as given in NICHD guideline. The algorithm for the classification is shown in Algorithm 2.
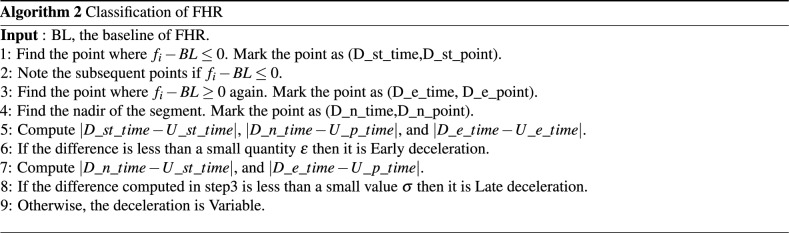


With this algorithm, 90 decelerations were identified from 125 CTG traces. This Classification using this feature set is performed using Random Forest, MLP, Naïve Bayes, and Simple Logistics using 5-fold cross-validation.

#### Warrick’s method

We have compared the outcome of our classification done with the feature sets given in the previous subsection with the method used by Warrick et al.^16^. They had identified the deceleration and the corresponding UCP. The feature set used by them was:

- I= D_st_time, D_n_time, D_e_time, U_st_time, U_p_time, U_e_time

The feature points were estimated using the definition mentioned in the NICHD literature. This feature set was used as an input to NN. The architecture of the NN consisted of 6 inputs, 4 hidden layers and a single output. The classification label was provided by:

- Annotation by clinicians by visual interpretation and the event points computed using the proposed method in Algorithm 1.
- Annotation by the crisp method and the event points computed using Algorithm 2.

### Ethical approval

This study used a secondary dataset which has been shared under the Open Data Commons Attribution License v1.0. As the data had already been anonymised, no ethical approval was required to perform the study.

## Results

In the absence of any ‘gold standard’, the obtained results as well as the performance of the classifiers were validated using several statistical measures.

### Inter-observer agreement

Inter-observer agreement was assessed both for the identification of deceleration, and the classification of deceleration.

#### Identification of deceleration

From the 125 traces considered in this study, 98 were found valid decelerations which the three clinicians agreed with. Clinician 1, Clinician 2, and Clinician 3 separately identified 103, 108, and 105 decelerations, respectively. NICHD guideline-based method identified 90 decelerations.

#### Classification of deceleration

**Figure 6:**
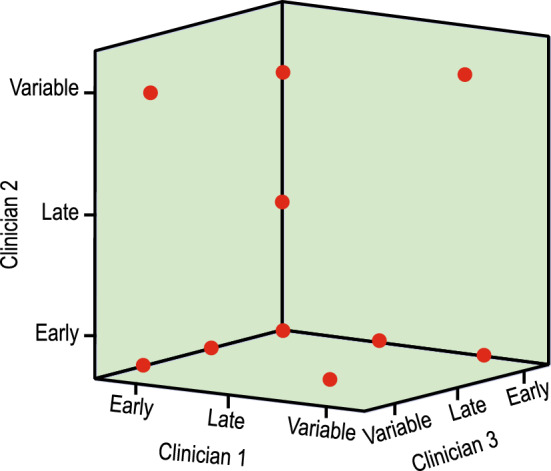
Agreement among the three clinicians in classifying the decelerations as Early, Late, and Variable.

The assessment of deceleration classification by each clinician is given in Table 6. The agreement between and among the clinicians was analysed using a single measure intra-class correlation coefficient (ICC). The difference between the standard correlation coefficient and ICC is that it is not dependent on the ordering of the data pairs. A two-way mixed model was used for inter-observer agreement. Table 7 shows the inter-observer single measure ICC of all the classes and the 95% CI as analysed by three clinicians. Agreement among the clinicians in classifying the decelerations is shown diagrammatically in Fig 6.

**Table 6:** Assessment of the classification of deceleration by the three clinicians.

Class	Clinician 1	Clinician 2	Clinician 3
Early	38	42	43
Late	21	20	18
Variable	38	35	36

**Table 7:** Intra-class correlation coefficient (ICC) for the agreement between the clinicians.

Class	ICC	95% CI	
		Upper limit	Lower limit
Early	0.988	0.981	0.985
Variable	0.984	0.953	0.962
Late	0.879	0.883	0.886

### Performance assessment of the classifiers

Evaluation of the different machine learning models for both feature sets is measured in terms of statistical parameters. Accuracy is a well-accepted metric to judge the performance of classifiers, however, the dataset has to be symmetric. Since, in the current experiment, the dataset is non-symmetric, we have also used the metrics such as True Positive (TP), False Positive (FP), precision, recall, F-score, and ROC. The results of all three classes using the four classifiers are shown in Table 8 for both sets of data. The confusion matrix for both sets is given in Table 9. Accuracy, kappa value, RMSE, and the other average statistical parameters of all the four classifiers for different classes are shown in Table 10.

**Table 8:** Statistical evaluation metrics of the classifiers for feature sets S1 and S2

Classifier	Statistical parameters of the classification for feature set S1								Class
	TP	FP	Prec.	Rec.	F-S.	ROC	Sen.	Spec.	
Random Forest	0.949	0.017	0.974	0.949	0.961	0.998	0.949	0.983	Early
	0.973	0.017	0.973	0.973	0.973	0.996	0.973	0.983	Variable
	1.0	0.013	0.955	1.0	0.977	1.0	1.0	0.987	Late
MLP	0.974	0.017	0.974	0.974	0.974	0.999	0.974	0.983	Early
	0.973	0.013	0.973	0.973	0.973	0.993	0.973	0.987	Variable
	1.0	0	1.0	1.0	1.0	1.0	0.999	0.998	Late
Naïve Bayes	0.872	0.017	0.971	0.872	0.919	0.989	0.872	0.983	Early
	0.919	0.133	0.810	0.919	0.861	0.938	0.919	0.867	Variable
	0.857	0.026	0.900	0.857	0.878	0.977	0.857	0.974	Late
Simple Logistics	0.949	0.017	0.974	0.949	0.961	0.987	0.949	0.983	Early
	0.973	0.050	0.923	0.973	0.947	0.986	0.973	0.950	Variable
	0.905	0.013	0.950	0.905	0.927	0.986	0.905	0.987	Late

Classifier	Statistical parameters of the classification for feature set S2								Class
	TP	FP	Prec.	Rec.	F-S.	ROC	Sen.	Spec.	
Random Forest	0.625	0.211	0.676	0.625	0.649	0.789	0.625	0.788	Early
	0.740	0.468	0.627	0.740	0.679	0.726	0.740	0.532	Variable
	0	0.011	0	0	0	0.464			Late
MLP	0.625	0.316	0.581	0.625	0.602	0.639	0.625	0.680	Early
	0.620	0.383	0.633	0.620	0.626	0.644	0.620	0.617	Variable
	0	0.056	0	0	0	0.483			Late
Naïve Bayes	0.450	0.193	0.621	0.450	0.522	0.646	0.48	0.785	Early
	0.660	0.511	0.579	0.660	0.617	0.591	0.66	0.49	Variable
	0	0.122	0	0	0	0.417			Late
Simple Logistics	0.600	0.263	0.615	0.600	0.608	0.668	0.66	0.737	Early
	0.600	0.404	0.612	0.600	0.606	0.598	0.61	0.61	Variable
	0	1.0	0	0	0	0.450			Late

TP: True Positive, FP: False Positive, Prec.: Precision, Rec.: Recall, F-S.: F-Score, ROC: Receiver Operating Characteristic Curve, Sen.: Sensitivity, Spec.: Specificity.

**Table 9:** Confusion matrix and MCC for feature set S1 and S2.

Confusion matrix for feature set S1					
	Early	Variable	Late		MCC
Random Forest	25	15	0	Early	0.4676
	12	37	1	Variable	
	0	7	0	Late	
MLP	38	1	0	Early	0.9647
	1	36	0	Variable	
	0	0	21	Late	
Naive Bayes	34	5	0	Early	0.8147
	1	34	2	Variable	
	0	3	18	Late	
Simple Logistics	37	1	1	Early	0.9129
	1	36	0	Variable	
	0	2	19	Late	

Confusion matrix for feature set S2					
	Early	Variable	Late		MCC
Random Forest	37	1	1	Early	0.9536
	1	36	0	Variable	
	0	0	21	Late	
MLP	25	13	2	Early	0.3743
	16	31	3	Variable	
	2	5	0	Late	
Naive Bayes	18	18	4	Early	0.3087
	10	33	7	Variable	
	1	6	0	Late	
Simple Logistics	24	14	2	Early	0.3469
	13	30	7	Variable	
	2	5	0	Late	

MCC: Matthews Correlation Coefficient for multiclass classification obtained using macro-averaging; calculated as: $MCC = \frac{TP*TN - FP*FN}{\sqrt{(TP+FP)(TP+FN)(TN+FP)(TN+FN)}}$.

**Table 10:** Accuracy of the classification by different classifiers for feature set S1 and S2.

Classifier	Statistical parameters of all the classifiers for feature set S1							
	Accuracy	Kappa	RMSE	Avg. TP	Avg. FP	Avg. Prec.	Avg. Recall	Avg. F-Score
Random Forest	96.91	0.952	0.974	0.969	0.016	0.969	0.969	0.969
MLP	97.94	0.968	0.81	0.979	0.013	0.979	0.979	0.979
Naïve Bayes	88.66	0.824	0.240	0.887	0.063	0.894	0.887	0.888
Simple Logistics	94.85	0.92	0.177	0.948	0.029	0.949	0.948	0.948

Classifier	Statistical parameters of all the classifiers for feature set S2							
	Accuracy	kappa	RMSE	Avg. TP	Avg. FP	Avg. Prec.	Avg. Recall	Avg. F-Score
Random Forest	63.92	0.317	0.398	0.639	0.329	0.602	0.639	0.618
MLP	57.73	0.236	0.490	0.577	0.332	0.566	0.577	0.571
Naïve Bayes	52.58	0.162	0.169	0.526	0.352	0.554	0.526	0.533
Simple Logistics	55.67	0.218	0.544	0.557	0.324	0.569	0.557	0.563

### Comparison of annotation by visual estimation with NICHD based estimation

The annotation of each trace given by the clinicians by visual estimation was compared with the crisp logic-based labelling given in NICHD guidelines.

#### Using ROC curve

**Figure 7:**
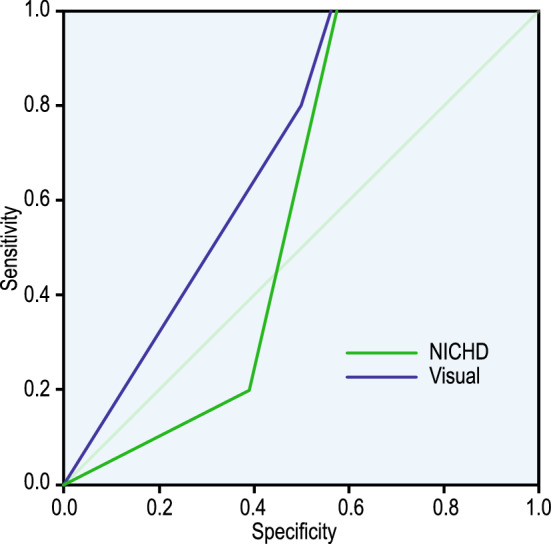
ROC curves for visual and NICHD-based estimation method from Table 11. AUC estimate for both visual and crisp logic-based classification.

The performance of each of the methods was done using a single measure, i.e., AUC under the ROC as shown in Fig. 7. The curves were plotted under the non-parametric assumption. The estimates of AUC are given in Table 11.

**Table 11:** AUC estimate for both visual and crisp logic-based classification

Test Result Variable(s)	Area	Std. Error	Asymptotic Sig.	Asymptotic 95% Confidence Interval	
				Lower Bound	Upper Bound
Visual	0.693	0.075	0.146	0.427	0.721
NICHD-based	0.574	0.085	0.579	0.526	0.861

#### Reliability measure using ICC

Both single measure and average measure ICC was used to compare the visual and crisp-based labelling of the decelerations. It was a two-way mixed effect model because the people effects were random and measure effects were fixed. The result is given in Table 12.

**Table 12:** Reliability measure using ICC

	Intraclass Correlation	95% Confidence Interval	
		Lower Bound	Upper Bound
Single Measures	0.766	0.670	0.838
Average Measures	0.868	0.802	0.912

#### Deming regression

**Figure 8:**
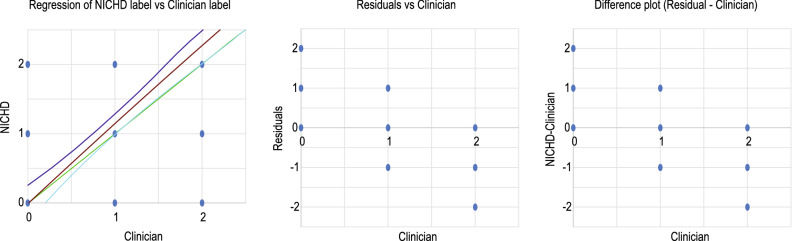
Left: Deming regression; middle: residual plot of clinician’s label; and right: difference plot of clinician’s label. The black line in the left subplot is the simple linear regression line, and the green line through the origin is the Deming regression fit line associated with a 95% confidence interval. The middle and right graphs show that the agreement between the methods is unsatisfactory.

The most common method of comparison of measurements is using linear regression (LR); however, it is done under the assumption that one of the measurements is error-free. The current study is not suitable for LR because none of the measurements is free of error. Hence, we have instead opted for Deming regression to compare the two methods. It is considered one of the best techniques for comparing methods when none of the methods is error-free. The model coefficient is given in Table 13, and the regression model with upper and lower bound of 95% CI, residual plot and the difference plot of clinician’s label are shown in Fig. 8.

**Table 13:** Model coefficient of Deming Regression

	Value	Lower bound 95% (Mean)	Upper bound 95% (Mean)
Intercept	0.018	$-0.117$	0.153
Slope coefficient	1.108	1.025	1.191

#### Bland-Altman Plot

**Figure 9:**
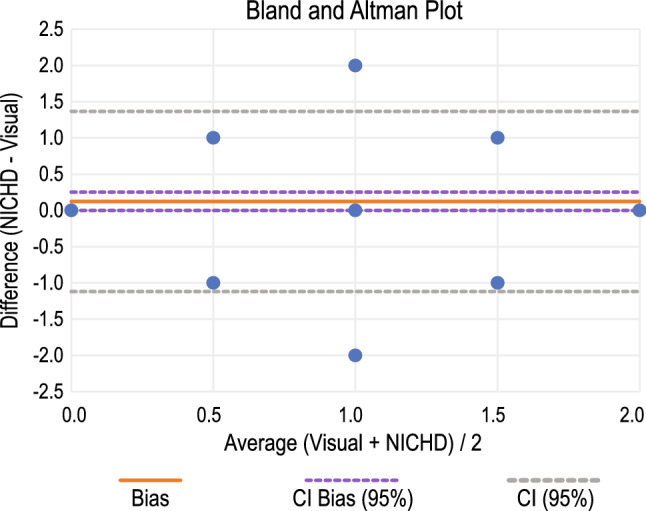
Bland-Altman plot with 95% CI for the comparison of visual annotation with NICHD guideline-based annotation.

Paired sample t-test yielded p > 0.05. The Bland-Altman plot, as in Fig. 9, was used for the two types of annotation method with a 95% confidence interval (CI). The mean value was found to be 0.2513, with the upper and lower limits of the agreement being 1.3644 and $-1.1167$, respectively.

### Statistical estimation of the classifier performance for NICHD-based annotation

Classifier model hyperparameters, statistical parameters associated with each classifier and the confusion matrix for the feature set S3 are given respectively in Tables 14, 15 and 16, respectively. The accuracy measures of the classifiers are given in Table 17.

**Table 14:** Classifier model hyperparameters.

Classifier	Hyperparameter	Values
Random Forest	Batch size	100
	Bag size	100
	Iterations	100
	Seed	1
MLP	Batch size	100
	Hidden layers	2
	Learning rate	0.4
Naïve Bayes	Batch size	100
Simple Logistics	Batch size	100
	Heuristic stop	50
	Max boosting iteration	400

**Table 15:** Statistical parameters of the classification annotated using NICHD guidelines

Classifier	TP	FP	Prec.	Rec.	F-S	ROC	Sen.	Spec.	Class
Random Forest	0.600	0.263	0.615	0.600	0.608	0.668	0.66	0.737	Early
	0.600	0.404	0.612	0.600	0.606	0.598	0.60	0.61	Variable
	0	1.0	0	0	0	0.450			Late
MLP	0.650	0.316	0.591	0.650	0.619	0.699	0.66	0.49	Early
	0.660	0.298	0.702	0.660	0.680	0.729	0.66	0.70	Variable
	0	0.067	0	0	0	0.656			Late
Naïve Bayes	0.450	0.193	0.621	0.450	0.522	0.646	0.45	0.80	Early
	0.660	0.511	0.579	0.660	0.617	0.591	0.66	0.49	Variable
	0	0.122	0	0	0	0.601			Late
Simple logistics	0.525	0.263	0.583	0.525	0.553	0.659	0.58	0.73	Early
	0.660	0.532	0.569	0.660	0.611	0.602	0.66	0.46	Variable
	0	0.033	0	0	0	0.368			Late

TP: True Positive, FP: False Positive, Prec.: Precision, Rec.: Recall, F-S: F-Score, ROC: Receiver Operator Characteristic, Sen.: Sensitivity, Spec.: Specificity.

**Table 16:** Confusion matrix of the classifiers with annotation using NICHD guidelines.

Classifier	Early	Variable	Late	Class
Random Forest	24	12	2	Early
	12	30	6	Variable
	1	3	0	Late
MLP	26	8	4	Early
	14	33	1	Variable
	2	2	0	Late
Naïve Bayes	18	16	4	Early
	9	33	6	Variable
	0	4	0	Late
Simple logistics	21	17	0	Early
	12	33	3	Variable
	0	4	0	Late

**Table 17:** Metrics for the performance evaluation of the classifiers when annotated using NICHD guidelines.

Classifier	Acc.	Kappa	RMSE	ATP	AFP	A. Prec.	A. Rec.	A. F-S
Random Forest	55.67	0.218	0.544	0.557	0.324	0.569	0.557	0.563
MLP	60.82	0.300	0.429	0.608	0.289	0.606	0.608	0.606
Naïve Bayes	52.58	0.162	0.490	0.526	0.352	0.554	0.526	0.533
Simple Logistics	55.67	0.174	0.441	0.557	0.385	0.534	0.557	0.543

Acc.: Accuracy, ATP: Average True Positive, AFP: Average False Positive, A. Prec.: Average Precision, A. Rec.: Average Recall, A. F-S: Average F-Score.

**Figure 10:**
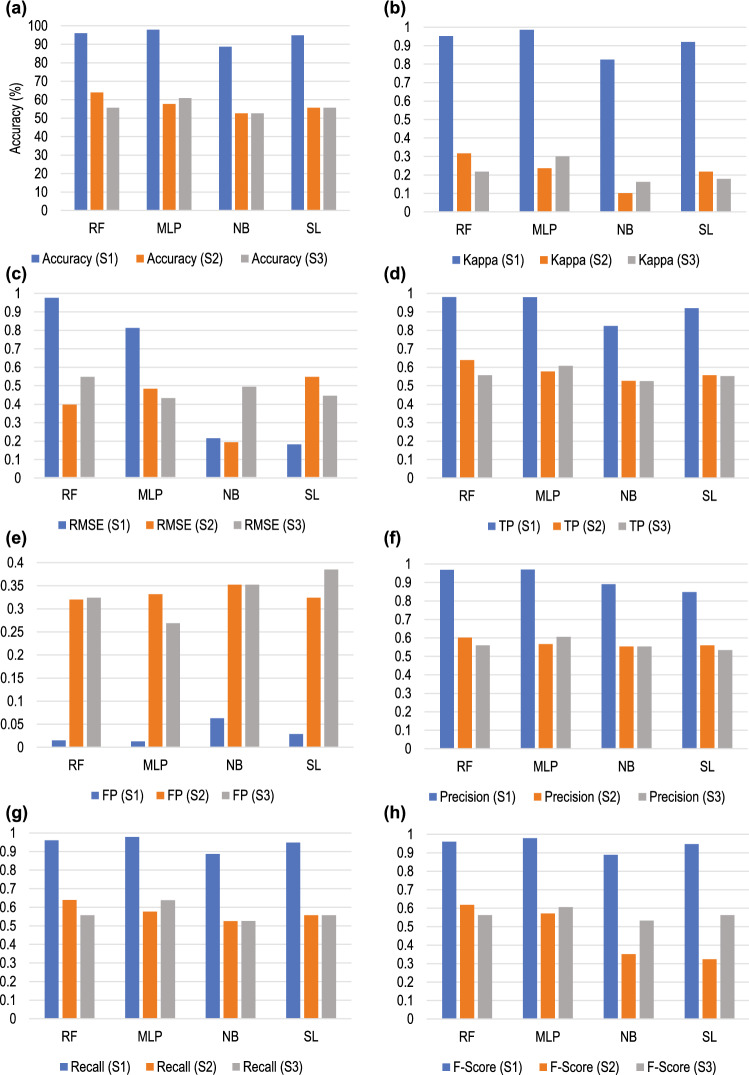
Comparison of different metrics of the classification of the three datasets denoted as S1, S2 and S3. The metrics are: (**a**) Accuracy; (**b**) Kappa; (**c**) RMSE; (**d**) True Positive (TP); (**e**) False Positive (FP); (**f**) Precision; (**g**) Recall; and (**h**) F-Score.

A comparison of the average measurement of matrices of the classification for the three datasets is shown graphically in Fig. 10.

### Statistical estimation of the neural network-based model of warrick

**Figure 11:**
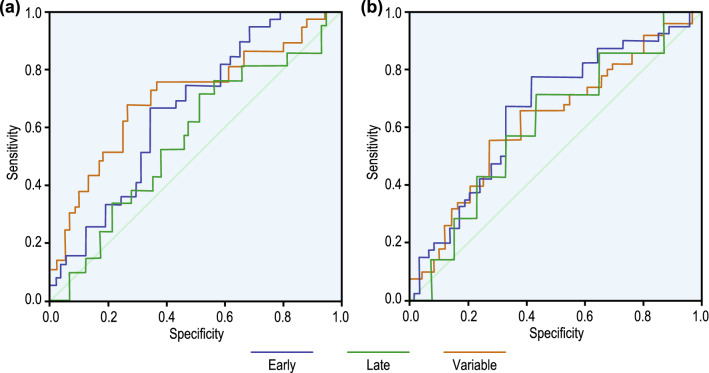
ROC for the NN-based classification with (a) training data label provided by the clinicians, and (b) training data label computed using NICHD definition. Since the late deceleration curve is approximately near the 45°  diagonal, the model is not very robust.

The accuracy of the NN-based classification with labels provided by the clinicians is given in Table 18, and the outcome with labels provided using NICHD-based classification is given in Table 19. ROC of the classification for both the data sets are given in Fig. 11, and the corresponding classification accuracy is given in Table 20.

**Table 18:** Accuracy of the classification by NN with the class label provided by the clinicians.

Sample	Observed	Predicted			Percent Correct
		Early	Late	Variable	
Training	Early	17	0	8	68.0%
	Late	9	0	7	0.0%
	Variable	9	0	21	70.0%
Testing	Early	9	0	5	64.3%
	Late	4	0	1	0.0%
	Variable	3	0	4	57.1%

**Table 19:** Accuracy of the classification by NN with the class label provided using NICHD guidelines.

Sample	Observed	Predicted			Percent Correct
		Early	Late	Variable	
Training	Early	15	0	14	51.7%
	Late	1	0	5	0.0%
	Variable	10	0	21	67.7%
Testing	Early	5	0	6	45.5%
	Late	1	0	0	0.0%
	Variable	7	0	12	63.2%

**Table 20:** Classification accuracy of each class for ROC (a) label by clinicians, (b) label using NICHD definition.

	(a) Label by clinicians		(b) Label using NICHD definition	
		Area		Area
Classified as	Early	0.659	Early	0.653
	Late	0.561	Late	0.604
	Variable	0.704	Variable	0.621

## Discussion

We have discussed the outcome for different experimental scenarios such as inter-observer agreement, classifiers performance assessment, visual vs NICHD-based classification, NN based model of Warrick in the following subsections:

### Inter-observer agreement

Table 6 shows considerably good agreement among the clinicians in classifying the deceleration. This is confirmed by the ICC $> 0.8$ in Table 7 for all three classes, indicating an excellent inter-rater agreement. Also, the difference between the upper and lower limits of agreement given by 95% CI is narrow for all three classes. The 3-D graphical representation of Fig. 6 shows very few outliers for the inter-rater agreement.

### Performance assessment of the classifiers

Accuracy as a metric may give a biased result. Performance assessment of the classifiers was based on the metrics TP, FP, Precision, Recall, F-score, and AUC of ROC as shown in Table 8. TP $> 0.95$ for all three classes when the feature set S1 was evaluated using MLP. FP, on the other hand, was in the range of 0–0.017 for all three classes when the classification algorithm used RF and MLP.

Precision, is a measure of the surety of TPs and Recall, is a measure of the surety that none of the positives was missed. In the scenario of foetal health assessment since the idea of FP is better than false negative (FN) and since it is necessary to be confident of TPs, we concentrated on the values of recall and precision respectively. Both these parameters had a value $> 0.95$ for dataset S1 when it was classified using MLP. F-score measures the accuracy of a model based on precision and recall. Thus, F-score is also $>0.97$ with MLP. AUC of ROC determines the optimum threshold value for classification. For feature set S1 the ROC was found to be $> 0.95$ for all the classifiers, however, ROC $=$ 1 was noticed for Late deceleration for RF and MLP.

The feature set S2 had TP, Precision, Recall, and F-score 0 for Late deceleration when classified with all the four classifiers, whereas, FP values were comparatively much higher. ROC $< 0.5$ for Late deceleration with all the classifiers.

Visualisation of the performance of the machine learning algorithms is given in the confusion matrix of Table 9. For feature set S1, MLP was able to accurately identify all three classes with FN $\simeq$ 0. Most significantly, all the late decelerations were correctly identified. For feature set S2, RF exhibited a good performance with FN $\simeq$ 0.

Analysis of the metrics for the average performance of the classifiers given in Table 10 reveals that for feature set S1 accuracy and kappa were highest with 97.94% and 0.968 respectively for MLP. For S2 the same metrics had values of 63.92% and 0.317 respectively with the RF classifier. A summary of the performance of each classifier for both the feature sets is given in Table 21.

**Table 21:** Summary of performance of different classifiers for feature sets S1 and S2 in terms of the parameter values.

Classifier	TP	FP	Precision	Recall	F-score	ROC	Accuracy	Kappa	FN	Feature Set
MLP	Yes	Yes	Yes	Yes	Yes	Yes	Yes	Yes	Yes	S1
RF	No	Yes	No	No	No	Yes	No	No	No	
Naïve Bayes	No	No	No	No	No	No	No	No	No	
Simple Logistics	No	No	No	No	No	No	No	No	No	
MLP	No	No	No	No	No	No	No	No	No	S2
RF	No	No	No	No	No	No	Yes	No	No	
Naïve Bayes	No	No	No	No	No	No	No	No	No	
Simple Logistics	No	No	No	No	No	No	No	No	No	

### Comparison of visual classification with NICHD-based classification

Comparing these two modes of assigning labels to a deceleration was done using the ROC curve, ICC, and Bland-Altman plot. The ROC curve is one of the most important metrics to visualise the trade-off between sensitivity and specificity. From Fig. 11, the AUC $> 0.5$ for both the curves; however, the AUC of NICHD classification below the diagonal has an FP rate higher than the TP rate. Generally, the sensitivity or the TP rate of visual interpretation is always higher than NICHD-based estimation.

ROC-AUC considers the c-statistics, which measures the probability that visual estimation discriminates better between the classes than the NICHD-based method^39^. However, it says nothing about the agreement between the two methods. ICC was used to measure the strength of agreement. The correlation value was found to be greater than 0.75 for both single and average measures, as shown in Table 11, indicating moderate to good agreement. This is not sufficient to suggest that these methods of labelling are the same since none of the methods is error-free.

Since both techniques contain error, Deming regression was used to fit a straight line to the two-dimensional data. In Fig. 8 left panel, the black line is the simple linear regression line and the green line through the origin is the Deming regression fit line associated with 95% CI. Based on these two lines and the residual plots in Fig. 8 (middle and right subplots) it can be concluded that the agreement between the methods is not satisfactory.

Before finding the degree of disagreement, it was necessary to check whether these two modes of classification could be used interchangeably, paired sample t-test was carried out, and it yielded p $> 0.05$, i.e., the null hypothesis could not be rejected, but that it could not be accepted either. We, thus, used the Bland-Altman plot which is given in Fig. 9. Though there is an insignificant number of outliers, most of the data points do not fall near the line of equality, and also, the limits of agreement are wide, indicating the existence of a significant degree of disagreement between the methods.

### Classifier performance for NICHD-based labelling

The true positive (TP) of all the classifiers for Late deceleration is zero, while the values for other metrics for the different classes of deceleration are not satisfactory. Confusion matrix of Table 15 that the performance of most of the failures to identify Late deceleration and the performance in identifying other types of deceleration is below average. The outcome reaffirms this inference in Table 16, which shows that the average measure of the different metrics for all four classifiers is below the acceptable limit.

### Performance of neural network based model of warrick

We verified the NN-based model proposed by Warrick using the label provided by the clinicians as well as the label assigned using NICHD-based guidelines. It is evident from Table 17 that for both the datasets, the model’s performance is average in identifying Early and Variable deceleration during the training and the testing phase. The model, however, completely failed to identify the Late deceleration. The ROC curve in Fig. 11a,b show that the late deceleration curve is closest to the 45$^\circ$ diagonal, indicating a lack of robustness of the model.

## Conclusion

A novel method for the classification of the deceleration of FHR has been proposed in this work. A fuzzy logic-based approach has been followed for estimating the length and width of the negative deviations from the baseline to identify the true deceleration. The event points of both FHR and the corresponding uterine contraction are computed. Two feature sets were used, each with 12 event points and the baseline of FHR. The first feature set (S1) consisted of event points calculated using the proposed algorithm. The second feature set (S2) consisted of event points and the baseline marked by the clinicians. For both feature sets, the identified decelerations were given a class label by the three expert clinicians after visual inspection. The performance of the different machine learning algorithms is summarised in Table 19. S1 had the highest accuracy of 97.94% with MLP, and S2 had the highest accuracy of 63.92% with Random Forest.

To establish the robustness of the proposed method, we used a third feature set (S3) which consisted of event points, baseline and the class label provided using strict NICHD guidelines. We have already established using statistical measures that the class label provided by the clinicians using visual estimates was better than the classification given by the crisp-logic-based NICHD method, and one cannot be replaced by the other. The result obtained after training different machine learning-based classifiers was unacceptable. Also, the number of decelerations identified using this crisp-logic-based approach was 8.16% less than the proposed approach.

The NN-based model of Warrick provided an accuracy of 70% when the clinicians provided the class label and around 55% when NICHD provided the label. This goes to show that the feature set used by Wariick’s model is not sufficient to provide the required level of accuracy. It can thus be concluded that we used the optimum feature set in the proposed method.

Since Late deceleration is an ominous pattern, its correct identification is a priority for any decision-making system. This was neither achieved with feature sets S2 and S3 nor with the NN-based model of Warrick. Also, NICHD-based identification and classification of deceleration are based upon crisp logic, which fails to identify the patterns in the grey zone.

## Data Availability

This study used a secondary dataset as described by Chudáček et al.^27^. The dataset can be obtained from the https://physionet.org/ repository using this direct link: https://physionet.org/content/ctu-uhb-ctgdb/1.0.0/.
